# AutoLock: a semiautomated system for radiotherapy treatment plan quality control

**DOI:** 10.1120/jacmp.v16i3.5396

**Published:** 2015-05-08

**Authors:** Joseph M. Dewhurst, Matthew Lowe, Mark J. Hardy, Christopher J. Boylan, Philip Whitehurst, Carl G. Rowbottom

**Affiliations:** ^1^ Christie Medical Physics & Engineering The Christie NHS Foundation Trust Manchester UK

**Keywords:** treatment planning, quality assurance, quality control, automation

## Abstract

A semiautomated system for radiotherapy treatment plan quality control (QC), named AutoLock, is presented. AutoLock is designed to augment treatment plan QC by automatically checking aspects of treatment plans that are well suited to computational evaluation, whilst summarizing more subjective aspects in the form of a checklist. The treatment plan must pass all automated checks and all checklist items must be acknowledged by the planner as correct before the plan is finalized. Thus AutoLock uniquely integrates automated treatment plan QC, an electronic checklist, and plan finalization. In addition to reducing the potential for the propagation of errors, the integration of AutoLock into the plan finalization workflow has improved efficiency at our center. Detailed audit data are presented, demonstrating that the treatment plan QC rejection rate fell by around a third following the clinical introduction of AutoLock.

PACS number: 87.55.Qr

## I. INTRODUCTION

In modern radiotherapy there is continuing emphasis on quality management to improve patient safety and reduce errors.^1^, ^2^, ^3^ A key component of radiotherapy quality assurance (QA) is independent checking of treatment plans.^(1)^ This quality control (QC) typically involves independent evaluation of plan quality and verification of technical and safety critical plan details, and has been found to be one of the most effective tools for error prevention.^(4)^ As radiotherapy moves towards more complex treatment delivery techniques, such as intensity‐modulated radiotherapy (IMRT) and volumetric‐modulated arc therapy (VMAT), the scope and complexity of treatment plan QC is increasing. The appropriate application of automation may be a useful tool to deal with some of these additional pressures while also reducing the potential for errors.^(5)^

Indeed, many elements of treatment plan QC are well suited to automation because they are time‐consuming and difficult for a human to perform but can be checked in a straightforward manner and quickly by computational procedures. Efforts to automate some aspects of treatment plan QC have developed in parallel with efforts to automate treatment planning itself. For example, Siochi et al.^(6)^ developed a system to automatically compare treatment planning system (TPS) data with record and verify system (R&V) data. Furhang et al.^(7)^ described a system that checks automatically the agreement between diagnosis, prescription, plan, and R&V values (intraplan review) and compares a treatment plan with other similar plans (interplan review). Yang and Moore^(8)^ developed a comprehensive system that checks automatically the physical and dosimetric integrity of treatment plans. Yang et al.^(9)^ described a system that automatically performs patient chart checking, and Halabi and Lu^(10)^ described a versatile system with the ability to parse TPS output PDF reports.

While many elements of treatment plan QC are objective and well suited to automation, some are more subjective or difficult to fully automate and better suited to human evaluation. In such cases, a checklist can be a useful tool. Checklists are an established tool in error management, and are increasingly being applied in the health‐care setting, following UK and international guidelines.^1^, ^2^, ^3^ For example, the clinical implementation of electronic checklists in radiotherapy has been described by Albuquerque et al.^(11)^ and Greenwalt et al.^(12)^ to improve patient safety and reduce errors. Breen and Zhang^(13)^ described the clinical implementation of an automated checklist for head‐and‐neck IMRT treatment planning. However, its effectiveness was limited by low compliance.

The ideal treatment plan QC system would accommodate both objective and subjective checks, apply comprehensively to the full breadth of treatment planning, and be fully integrated into the treatment planning workflow. In this paper, we describe the development and clinical implementation of a semiautomated system designed for this purpose, named AutoLock. AutoLock is intended to augment treatment plan QC by automatically checking aspects of treatment plans that are well suited to computational evaluation, whilst summarizing more subjective aspects in the form of a checklist. The principle is to automate as much as possible, while also acting as a filter to condense the large amount of information requiring human evaluation into a manageable checklist. The treatment plan must pass all automated checks, and all checklist items must be actively acknowledged by the planner as correct before the plan is finalized. Thus AutoLock uniquely integrates automated treatment plan QC, an automated checklist, and plan finalization.

In addition to reducing the potential for the propagation of errors, the integration of AutoLock into the plan finalization workflow is intended to improve efficiency at our center. By performing automated checks as part of treatment plan finalization, any errors detected can be corrected at a point in the workflow where this is straightforward to do so — such as before the plan is finalized by the planner — thereby reducing the downstream workload. With this in mind, we present detailed audit data demonstrating a reduction in the rate of treatment plan QC rejections following the clinical introduction of AutoLock.

## II. MATERIALS AND METHODS

### A. Treatment planning workflow

**Figure 1:**
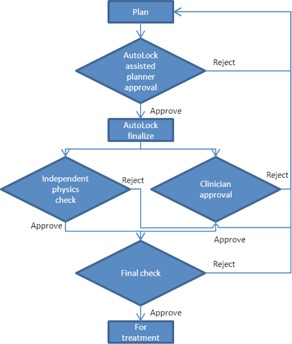
Simplified treatment planning workflow at our center and the role of AutoLock within it.

Figure 1 shows a simplified version of the AutoLock‐assisted treatment planning workflow and review process at our center. AutoLock is used by the planner to review, approve and finalize the treatment plan before it is independently checked and reviewed by the clinician. AutoLock is positioned as far upstream in the workflow as possible to help avoid the propagation of errors.

### B. Program design

AutoLock is written in Java and integrated into the Philips Pinnacle^3^ TPS using Pinnacle scripting (Philips Healthcare, Andover, MA). (See Yang and Moore^(8)^ for a detailed discussion of a similar technique.) Pinnacle scripting is a powerful feature of the TPS, which allows sequences of user interactions to be automated. In addition, external UNIX commands can be executed, allowing Pinnacle scripts to be combined with a high‐level programming language such as Java. The general principle behind AutoLock is that the scripting functionality of Pinnacle is used to store plan information in a text file; this plan information file is then processed independently of the TPS using logic statements written in Java. Check tolerances and settings are stored in a separate configuration file. In principle, this technique could be applied to any TPS with suitable scripting capabilities.

**Figure 2:**
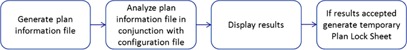
AutoLock Review module workflow.

AutoLock is split into two separate modules: Review and LockPlan. The Review module generates a plan information file, performs checks using this file in conjunction with the configuration file, and displays the check results to the user (see Fig. 2). If the results are then accepted by the user, the details of the review process are stored in a temporary HTML file called the “Plan Lock Sheet”.

**Figure 3:**
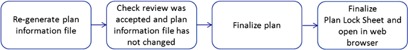
AutoLock LockPlan module workflow.

Once a plan has been reviewed and accepted by the planner, the LockPlan module is used to finalize the plan. The LockPlan module first checks that the plan has not changed since successful review, by regenerating the plan information file and comparing a checksum generated using this file with a checksum generated using the original plan information file. If changes are detected, the user is prompted to rerun the Review module. If no changes are detected, the plan and HTML Plan Lock Sheet are finalized (see Fig. 3), or “locked”. The Plan Lock Sheet acts as a permanent record of the planner review and finalization process and can be retrieved as necessary — for example, during independent checking of the plan.

### C. Check result taxonomy

Plan check results can be “Pass”, “Fail”, “For review”, “Not checked”, or “Error” (see Table 1). Each result is accompanied by a textual description, which explains the details of the result to the user.

**Table 1:** AutoLock check result taxonomy and meanings.

*Result*	*Meaning*
Pass	The check has passed
Fail	A problem has been detected with the treatment plan
For review	No specific problems have been detected but the result must be reviewed and acknowledged as correct by the user
Not checked	The check is not required for the current treatment plan
Error	A programming error has occurred

“Pass” and “Fail” results occur only where a check can be fully automated, and subjectivity is handled with “For review”. “For review” results occur where the check cannot be fully automated or human evaluation is required; a summary of such results is automatically added to a checklist for the planner to review. To allow full flexibility in the system, “Fail” results can also be acknowledged and thereby overridden by the planner. The details of an AutoLock check can vary depending on the type of plan, and in some cases a check may not apply, in which case a “Not checked” result occurs. “Pass” and “Not checked” results do not require user acknowledgment.

As stated above, the general principle applied in AutoLock is to automate as much as possible (i.e., maximize the number of “Pass” and “Fail” results and minimize the use of “For review”). It should be noted that a “For review” result does not necessarily imply that no automation has occurred; rather, as much automation as possible has occurred. In this way, AutoLock effectively acts as a filter to condense the large amount of information requiring human evaluation into a manageable checklist.

For completeness, the possibility of an “Error” result is included and indicates an unexpected programming event. As with “Fail” results, “Error” results can be overridden by the planner to allow flexibility but would, in practice, be thoroughly investigated before proceeding any further. All check results and acknowledgements appear on the Plan Lock Sheet, which can be reviewed during independent checking.

### D. User interface

**Figure 4:**
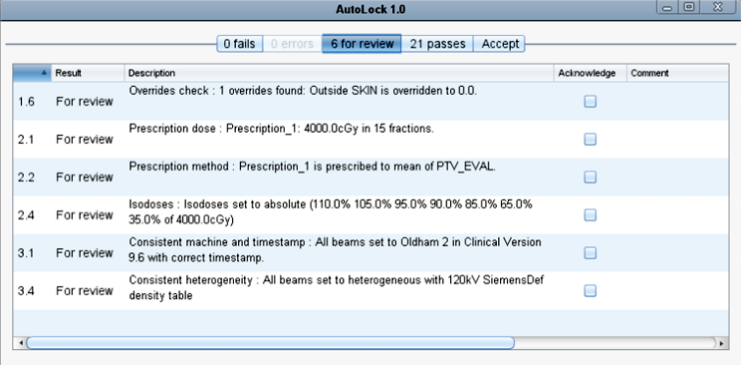
Screenshot of the “For review” tab in the AutoLock graphical user interface; each checklist item must be acknowledged by the user by completing a tick box.

Figure 4 shows a screenshot of the AutoLock graphical user interface (GUI), which appears to the planner upon running the AutoLock Review module. Results are split into tabs depending on the check result; Fig. 4 shows the “For review” tab. Any result requiring acknowledgement is acknowledged by completing a tick box. Comments can be added to each item as required by the planner and appear on the Plan Lock Sheet.

**Figure 5:**
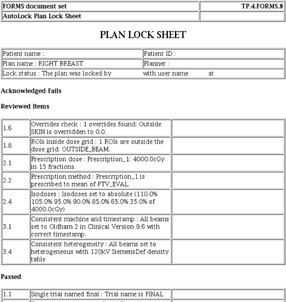
Extract from the Plan Lock Sheet, which shows all results of the plan review, split by result type.

Only once all items requiring acknowledgement have been acknowledged can the review be accepted in the “Accept” tab. This data completeness check means an active response, in the form of completing tick boxes, is required before the review can be accepted. Once the review has been accepted, the plan can be finalized using the LockPlan module. Figure 5 shows an example of the Plan Lock Sheet; as with the GUI, results are organized depending on the check result.

### E. Checks

Plan checks have been split up into discrete checks, which are given a unique number and assigned to one of four categories: 1) General plan checks; 2) Prescription checks; 3) Beam checks; 4) Control point checks.

There are currently 31 checks implemented in AutoLock, which includes checks of prescription, dose calculation parameters, density overrides, beam labeling, and MLC/jaw positions (see 2, 3, 4, 5). The implemented checks were chosen based on a review of practice, an audit of treatment plan QC rejection reasons, a review of the literature,^6^, ^7^, ^8^, ^9^, ^10^, ^13^ and a survey of checkers. It should be noted that many of the checks are tailored specifically to working practice at our center with the Pinnacle TPS. However, AutoLock is designed as an expandable model and, therefore, more or different checks can be added in a straightforward manner.

**Table 2:** AutoLock general plan checks.

*ID*	*Name*	*Description*
1.1	Single trial named final	Plan has only one trial and is labeled correctly
1.2	Couch removed	Couch is removed (does not check couch removal position)
1.3	Dose grid voxel size 0.3 cm or less	Dose grid voxel size is set appropriately
1.4	Air threshold is $0.81\;g/{\text{cm}}^{3}$ or less	Patient–air threshold is set appropriately
1.5	Origin and isocenter POI check	Origin POI matches localizer, origin and isocenter POIs are correctly formatted
1.6	Overrides check	Overrides are set appropriately; a list of overrides present is displayed for the user to review
1.7	Plan name length	Plan name length is less than required for DICOM export
1.8	ROIs inside dose grid	All ROIs are covered entirely by dose grid (exceptions can be set in the configuration file)
1.9	Has the MU checker run?	For 3D conformal plans, prompts the planner to check the plan runs through the independent monitor unit checking software
1.10	Plan comment contains final	Plan is labeled correctly
1.11	Correct version of Pinnacle	Current clinical version of Pinnacle is in use

**Table 3:** AutoLock prescription checks.

*ID*	*Name*	*Description*
2.1	Prescription dose	Prescription options are set correctly; the prescription dose and number of fractions are displayed for the user to review
2.2	Prescription method	Correctly prescribed to point dose or ROI mean (and not monitor units); the prescription point or ROI is displayed for the user to review
2.3	Weights proportional to	Beam weights are correctly set proportional to monitor units or point dose
2.4	Isodoses	Isodose lines set appropriately; a list of isodoses present is displayed for the user to review
2.5	Max point dose less than or equal to 110%	Flag for potential hotspot issues

**Table 4:** AutoLock beam checks.

*ID*	*Name*	*Description*
3.1	Consistent machine and timestamp	All beams are set to the same beam model and timestamp matches the quality system; the beam model in use is displayed for the user to review
3.2	Consistent beam types	All beams are set to the same type (e.g., conventional, IMRT or VMAT)
3.3	All beams set to AC or CCC	All beams are set to either adaptive convolve or collapsed cone convolution
3.4	Consistent heterogeneity	All beams are set to heterogeneous or homogeneous and appropriate CT‐density table is in use
3.5	All beams set to consistent isocenter	All beams set to the same isocenter
3.6	All beams set to consistent prescription	All beams set to the same prescription unless multiple prescriptions are present, in which case the user must review
3.7	All beams have a field ID entered	All beams have a field ID entered to enable unique identification in the R&V system, regardless of beam name
3.8	Beam labels check	Beam labels are correct
3.9	In vivo points check	*In vivo* dosimetry POIs have been generated correctly (name, at depth $d_{\text{max}}$, and position within field)

**Table 5:** AutoLock control point checks.

*ID*	*Name*	*Description*
4.1	No control points with less than 3 MUs	No control points have less than the minimum 3 monitor units
4.2	Y jaw backup	Y jaws are backed up correctly
4.3	X jaw backup	X jaws are backed up correctly
4.4	Elekta breast IMRT back edge check	Back edge jaw position is consistent for Elekta breast IMRT plans
4.5	No blocks present for plans not using MLC	No blocks are present for plan not using the MLC
4.6	Varian CAX matchlines	No matchlines present near the central axis for Varian step‐and‐shoot IMRT plans

### F. Audit

#### F.1 AutoLock log file audit

AutoLock log files were collected for plans produced in a six‐month period since clinical introduction at our center. Changes in check results between the first time AutoLock was run and last time AutoLock was run were counted. Any check result changing from “Fail” to “Pass”, “Fail” to “For review”, or “For review” to “Pass” was considered to indicate a positive AutoLock prompted action. The percentage action rate was calculated over all plans, on a month‐by‐month basis, and for each check individually.

#### F.2 Treatment plan QC rejection audit

To assess the broader impact of AutoLock on the workflow, treatment plan QC rejections were compared before and after clinical introduction. At our center, each time a treatment plan is rejected at the independent checking stage, it is assigned a code indicating the reason for audit purposes (see Table 6). These rejection codes were compared before and after the clinical introduction of AutoLock. The first month of audit data following the introduction of AutoLock was excluded from the analysis to avoid the initial ramp‐up phase. The remaining five months of data were compared with the five months immediately prior to the introduction of AutoLock.

**Table 6:** Thirteen‐category treatment plan QC rejection coding system.

*ID*	*Name*	*Description*
1	Contouring	ROI delineation and expansion
2	Density correction	Density overrides
3	Documentation	Missing or incorrect information in plan printout
4	Dose calculation	Beam model, dose calculation, voxel grid size, couch removal
5	InVivo points	*In vivo* dosimetry POIs
6	Jaw/MLC	Jaw backup, control point constraints
7	Modification of treatment by clinician	Patient setup, ill‐fitting shell, changes to patient, PTV, etc.
8	Optimization	Coverage, OAR doses, beam energy, shielding
9	Origin	Localization
10	Prescription	Prescription and method
11	Wedge angle	Wedge angle not permitted in quality system
12	Wrong linac	Beam model does not match linac booking
13	Other	Reason not described in system; recurrent reasons can be reviewed for inclusion via an additional category

## III. RESULTS

**Figure 6:**
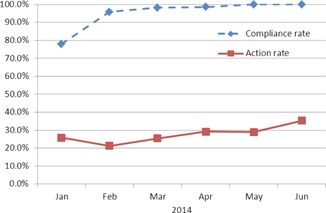
AutoLock compliance rate and action rate as a function of time, showing usage increased to 100% over the audit period and an upwards trend in action rate.

AutoLock was commissioned and introduced into routine clinical use at our center in January 2014. The commissioning process involved independent testing using a suite of test data containing deliberate errors, independent code verification, independent beta, and usability testing and training. Over the period of our audit, AutoLock was used to finalize 2,384 Pinnacle treatment plans, out of a total of 2,487 (i.e., 96%). Of the 2,384 plans finalized with AutoLock, 1,585 were 3D conformal or forward‐planned IMRT and 799 were inverse‐planned IMRT or VMAT. The majority of plans finalized without AutoLock were produced during January and February, during the initial ramp‐up phase. Compliance from March onwards was close to 100%, as demonstrated by the dashed line in Fig. 6.

**Figure 7:**
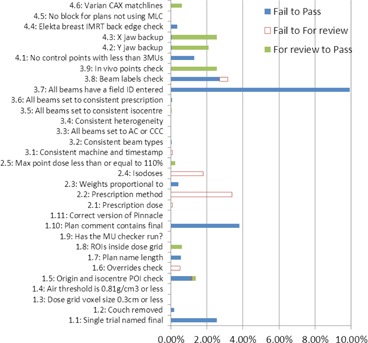
AutoLock action rates for individual checks.

The overall action rate for all plans finalized with AutoLock was 28%. The action rate was 27% for 3D conformal and forward‐planned IMRT and 30% for inverse‐planned IMRT or VMAT. The solid line in Fig. 6 shows the action rate over all plans finalized with AutoLock on a month‐by‐month basis. Figure 7 shows the action rates for each individual check over all plans finalized with AutoLock.

**Figure 8:**
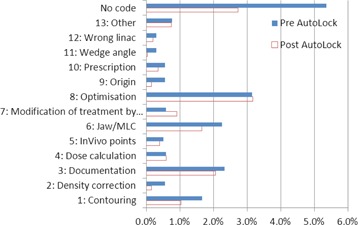
Treatment plan QC rejection codes before and after the introduction of AutoLock.

Figure 8 shows rejections code rates before and after the introduction of AutoLock, as well as rejections not assigned a rejection code. The overall treatment plan QC rejection rate was 32% lower after the introduction of AutoLock.

## IV. DISCUSSION

The results in 6, 7, 8 represent a fascinating insight into the impact of AutoLock, as well as the relative frequency of issues encountered during treatment plan QC. Figure 6 shows that the compliance rate rose to 100% over the period of our audit, considerably higher than achieved in the study by Breen and Zhang.^(13)^ This may be explained by a number of factors. Firstly, AutoLock is fully integrated into the treatment planning workflow and acts as the finalization process. This integration has helped AutoLock to quickly become established as routine treatment planning practice; indeed, since performing this audit, finalization with AutoLock has become mandated in our quality management system. Secondly, anecdotal evidence suggests that AutoLock has become a popular tool amongst planners as it is highly user‐friendly and provides instant, impersonal feedback. User friendliness was a key consideration in the design of AutoLock, as it is vital to the safe and effective use of software.^(14)^

Figure 6 also demonstrates a general upwards trend in the action rate, which may be explained by two factors. Firstly, AutoLock may be run at any point during the planning process and, therefore, the first time AutoLock is run, may not correspond to when a planner is ready to finalize a plan. The rising action rate suggests that planners may be running AutoLock earlier in the planning process. A second possibility is that planners familiar with AutoLock may have started to use it to catch issues that they would have checked manually had AutoLock been unavailable. In either case, this is suggestive of a workflow improvement, as planners may well be spending less time manually checking treatment plan details and instead utilizing the automation provided by AutoLock. Quantification of time savings for planners and checkers will be tackled with future work.

Figure 7 shows that action rates vary considerably between different plan checks. It should be noted that these action rates are likely to represent significant overestimates of the planning error rates that would have occurred had AutoLock not been available, for the same two reasons described in the previous paragraph. Nevertheless, these results suggest that AutoLock has had a positive impact at our center by prompting planners to correct issues before plan finalization. This is supported by the observed 32% reduction in the rate of treatment plan QC rejections following the introduction of AutoLock; a similar reduction was found in by Breen and Zhang.^(13)^

The impact of AutoLock would be further appreciated by showing a correlation between the results in Fig. 7 and Fig. 8 (i.e., between AutoLock action rates and QC rejection reasons). However, this is complicated by a number of issues. Firstly, a significant fraction of QC rejections have not been assigned a rejection code. Secondly, some AutoLock checks apply to multiple rejection codes and vice‐versa, and in some cases the correspondence is only partial. For example, check number 1.2 checks that the couch has been removed but does not check the position of the couch removal, which is left for human evaluation. The effects of this check would be observed in rejection category 4 (dose calculation). However, this category also encompasses a number of other AutoLock checks including 1.3, 1.4, 1.8, 3.1, 3.2, and 3.3 and there is no apparent reduction in the rate of rejections for this category.

However, there is a more direct correspondence with other categories. For example, in category 2 (density correction) a rejection rate reduction of around 70% was observed. This category closely corresponds to check number 1.6 which, amongst other things, displays the overrides present in the plan for the user to review. A rejection rate reduction of around 70% was also observed for category 9 (origin), which closely corresponds to check number 1.5, which checks coincidence of the origin POI and localizer, while positioning of the localizer is left for human evaluation. Smaller reductions in rejection rates occur in all other categories where AutoLock would be expected to have an impact: 3 (documentation), 5 (InVivo points), 6 (jaw/MLC), 10 (prescription), and 12 (wrong linac). These results will direct the future development of AutoLock checks and the rejection coding system as part of the continuous cycle of quality improvement at our center.

Aside from these results, many of the benefits of AutoLock are difficult to measure. For example, automated systems like AutoLock are particularly suited to catching infrequent errors that a human checker may miss on account of their infrequency. Indeed, a number of AutoLock checks showed an action rate of or very close to 0%. Nevertheless, there is still value in including such checks, particularly as the only cost to the department is in the initial development and commissioning process; every subsequent automated check is effectively “free” to the department.

Many of the checks implemented in AutoLock are designed specifically to improve workflow at our center. For example, the check with the highest action rate — checking that all beams have a “field ID” entered — is a relatively minor issue, but from a workflow point of view avoids potential downstream issues when the plan is imported into the R&V system. Smoothing the workflow is a key benefit of AutoLock because many small plan details can be checked automatically before plan finalization, thus increasing the consistency of treatment plan generation. Furthermore, one would expect that the reduction of variability will lead to greater prospects for quality improvement. Indeed, the intention behind AutoLock is to automate as much treatment plan QC as possible, and thereby allow the planner and checker to focus their attention on overall treatment plan quality and those safety critical details that are difficult or impossible to fully automate. More work is required to investigate whether a similar approach can be beneficial in areas of the complex radiotherapy process other than treatment planning.

## V. CONCLUSIONS

We have developed and clinically implemented a semiautomated system for treatment plan QC, named AutoLock. AutoLock is designed to augment treatment plan QC by automatically checking aspects of treatment plans that are well suited to computational evaluation, whilst summarizing more subjective aspects in the form of a checklist. The treatment plan must pass all automated checks and all checklist items must be acknowledged by the planner as correct before the plan is finalized. Thus AutoLock uniquely integrates automated treatment plan QC, an electronic checklist, and plan finalization.

AutoLock provides instant, impersonal feedback to the planner and acts as a filter to condense the large amount of information requiring human assessment into a manageable checklist. In addition to reducing the potential for propagation of errors, the integration of AutoLock into the plan finalization workflow is intended to improve efficiency at our center. AutoLock has been in clinical use at our center since January 2014 and the treatment plan QC rejection rate fell by around a third following its introduction.
